# Novel Approaches to Enzyme-Based Electrochemical Nanobiosensors

**DOI:** 10.3390/bios13060622

**Published:** 2023-06-05

**Authors:** Nur Melis Kilic, Sima Singh, Gulsu Keles, Stefano Cinti, Sevinc Kurbanoglu, Dilek Odaci

**Affiliations:** 1 Faculty of Science Biochemistry Department, Ege University, 35100 Bornova, Turkey; nurmeliskilic@gmail.com (N.M.K.); dilek.demirkol@yahoo.com (D.O.); 2 Department of Pharmacy, University of Naples Federico II, 80138 Naples, Italy; sima.singh@unina.it (S.S.); stefano.cinti@unina.it (S.C.); 3 Department of Analytical Chemistry, Faculty of Pharmacy, Ankara University, 06560 Ankara, Turkey; kelesgulsu@gmail.com

**Keywords:** nanobiosensors, biosensors, enzyme, substrate, electrochemistry

## Abstract

Electrochemistry is a genuinely interdisciplinary science that may be used in various physical, chemical, and biological domains. Moreover, using biosensors to quantify biological or biochemical processes is critical in medical, biological, and biotechnological applications. Nowadays, there are several electrochemical biosensors for various healthcare applications, such as for the determination of glucose, lactate, catecholamines, nucleic acid, uric acid, and so on. Enzyme-based analytical techniques rely on detecting the co-substrate or, more precisely, the products of a catalyzed reaction. The glucose oxidase enzyme is generally used in enzyme-based biosensors to measure glucose in tears, blood, etc. Moreover, among all nanomaterials, carbon-based nanomaterials have generally been utilized thanks to the unique properties of carbon. The sensitivity can be up to pM levels using enzyme-based nanobiosensor, and these sensors are very selective, as all enzymes are specific for their substrates. Furthermore, enzyme-based biosensors frequently have fast reaction times, allowing for real-time monitoring and analyses. These biosensors, however, have several drawbacks. Changes in temperature, pH, and other environmental factors can influence the stability and activity of the enzymes, affecting the reliability and repeatability of the readings. Additionally, the cost of the enzymes and their immobilization onto appropriate transducer surfaces might be prohibitively expensive, impeding the large-scale commercialization and widespread use of biosensors. This review discusses the design, detection, and immobilization techniques for enzyme-based electrochemical nanobiosensors, and recent applications in enzyme-based electrochemical studies are evaluated and tabulated.

## 1. Electrochemical Biosensors

**Figure 1:**
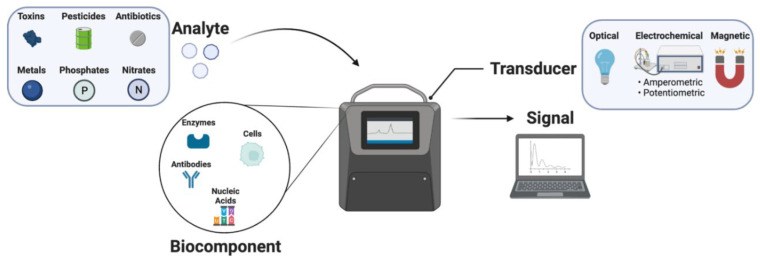
Schematic representation of electrochemical biosensors.

Electrochemistry is the study of a chemical species’ reaction at the interface of an electron conductor and an ionic conductor, where the charge is transported between the substances and the electrode. The electrode is often made of metal or a semiconducting substance, and the ionic conductor, known as the electrolyte, can be solution-based (dissolved ions in aqueous or organic solvents) or solid-based [1]. The discipline of electrochemistry is an important area of modern research that has brought together the sciences of electricity and chemistry. Electrochemistry is a genuinely interdisciplinary science that may be used in a wide range of physical, chemical, and biological domains [2]. Quantifying biological or biochemical processes is critical in medical, biological, and biotechnological applications. Nowadays, several electrochemical biosensors exist for various healthcare applications, such as for the determination of glucose, lactate, catecholamines, nucleic acid, uric acid, and so on [3]. A biosensor is a type of analytical equipment that can detect a specific analyte. Electrochemical sensors have been completely developed for several decades since the original concept of the biosensor was introduced in 1962, due to their excellent sensitivity and specificity [4]. The discipline is a multidisciplinary study area that connects basic science concepts (physics, chemistry, and biology) with micro/nanotechnology, electronics, and applied medicine foundations. In 2023, the database ‘Web of Science’ indexed around 90,000 reports on the topic of ‘biosensors’. Devices that detect analytes employing optical, piezoelectric, and electrochemical transducers have advanced dramatically during the last 50 years [5]. Biosensors are used in applications such as illness monitoring, drug development, detecting contaminants, analysis of disease-causing microorganisms, and identification of disease markers in physiological fluids (blood, urine, saliva, sweat). Figure 1 depicts a conventional biosensor comprising the following components: analyte, bioreceptor, transducer, and signal.

The analyte is the material that must be identified. A bioreceptor is a molecule that identifies the analyte specifically. Bioreceptors include enzymes, cells, aptamers, deoxyribonucleic acid (DNA), and antibodies [6,7,8,9,10]. Bio-recognition refers to the signal creation process (heat, pH, charge, mass change, etc.) that occurs when a bioreceptor interacts with an analyte. A transducer is a component that transforms one type of energy into another. The transducer in a biosensor converts the bio-recognition event into a quantifiable signal. This energy conversion process is called signalization. The majority of transducers provide optical or electrical signals that are proportional to the number of analyte–bioreceptor interactions [11]. Electrochemical biosensors, whose working principles are based on the electrochemical characteristics of the analyte and the transducer, are the most commonly researched and utilized biosensors. Electrochemical biosensors have excellent sensitivity, selectivity, and detection capabilities. An electrochemical interaction between the bioreceptor and the analyte happens on the transducer surface in this biosensor, providing visual electrochemical signals in the form of voltage, current, impedance, and capacitance [12]. Based on their transduction principle, electrochemical biosensors are categorized as potentiometric [13], amperometric [14], impedimetric [15], conductometric [16], voltammetric [17], and thermal [18]. However, translating biological material to a quickly processed electrical signal is difficult due to the intricacy of attaching an electronic device directly to a biological system. Due to the immediate transformation of a biological event to an electrical signal, electrochemical biosensors can provide an appealing technique for analyzing the content of a biological sample. Several sensing ideas and associated technologies have been developed over the last decades [19]. Electrochemical biosensors are analytical devices that convert biochemical events such as enzyme–substrate reactions and antigen–antibody interactions into electrical signals such as current, voltage, impedance, etc. [20]. Voltammetry is an electro-analytical approach that obtains information about an analyte by altering a voltage and measuring the resultant current. As a result, it is an amperometric method. Voltammetry comes in various forms, since there are several ways to modify a potential [19].

Because of their outstanding performance, mobility, simplicity, and low cost, electrochemical biosensors are starting to be employed in various analytical, medical diagnostics, and screening applications. In addition to biosensor advancements, portable analyzers for metabolites and electrolytes have been created [21]. The presence of biomolecules can cause changes in the refractive index near the conductive thin-film surface, such as that of nanofibers, for use as a biosensor [22]. Surface Plasmon Resonance (SPR) biosensors are one type of biosensor that may be used to determine a variety of surface binding interactions, such as small-molecule adsorption [23], protein adsorption on self-assembled monolayers [24], antibody–antigen binding [25], protein–DNA interactions [26], binding kinetics, affinity, equilibrium constants in sensing and more.

Electrochemical methods, such as Cyclic Voltammetry (CV), Differential Pulse Voltammetry (DPV), and Electrical Impedance Spectroscopy (EIS), allow for the in situ control and monitoring of such redox processes, as well as of electrical system responsiveness and reaction reversibility [27]. CV entails applying a potential to an electrode and measuring the current response. Typically, the experiment begins with the electrode at a low potential, which is gradually increased in a linear sweep toward a more positive value. This cyclic procedure is repeated in the opposite direction to finish the cycle. CV is useful for determining oxidation and reduction potentials, as well as the kinetics and mechanisms of electron transport processes at the electrode–electrolyte interface. It is widely used in a variety of applications, including analytical chemistry, materials research, and the creation of electrochemical sensors [28].

DPV is a sensitive electrochemical method for determining analyte species concentrations in solution. The current response of a biological or chemical process to an applied potential difference is measured by DPV. It is a pulsed voltammetric method that measures a current responsiveness at predefined intervals using brief potential pulses. Because of its capacity to distinguish between anodic and cathodic peaks, this approach may be utilized for both qualitative and quantitative analyses, providing information on the redox processes occurring during the measurements. DPV has grown in popularity because of its low detection limits, varied variety of approaches, and ease of usage [29].

EIS is a powerful technique used to estimate the electrical impedance of a material or system over a range of frequencies in a variety of domains, such as electrochemistry, material science, and biology. EIS measurements are made by introducing a modest alternating current voltage to the studied system and measuring the ensuing current response. The frequency-dependent information received by EIS measurements provides useful insights into the system’s electrical features, such as its complex resistance and capacitance. This technology offers several advantages, including its high sensitivity, non-invasive nature, and capacity to examine a wide range of materials and systems. EIS has found widespread use in sectors such as biological sensing and energy science [30].

Optical methods, on the other hand, such as optical waveguide light mode spectroscopy, SPR, and ellipsometry, are well recognized for their ability to assess mass adsorption kinetics. Gravimetric techniques, such as quartz crystal microbalance with dissipation monitoring), and imaging techniques, such as Atomic Force Microscopy (AFM) and fluorescent microscopy, such as confocal laser scanning microscopy, can also be successfully combined with electrochemical methods to improve the understanding of bio-interfacial phenomena. These approaches allow for excellent sensitivity near the transducing element’s surface (electrode, waveguide, tip, etc.) [31]. The high interfacial sensitivity shared by electrochemical and other forms of biosensors allows for the simultaneous extraction of a richer collection of initial data, as well as for enhanced control over the sensing environment [19]. Biosensors are also tiny and portable, enabling portable sensing devices to monitor on site effluents [32]. With the range of bio-recognition components (containing enzymatic, immunochemical, and non-enzymatic receptors), whole DNA elements and molecularly imprinted polymer (MIP) biosensors can be classified.

## 2. Enzymatic Biosensors

Enzymes are massive, complex macromolecules, mostly consisting of proteins, that catalyze the quick transformation of substrates into products [33]. Enzymes are the prototypical examples of catalytic molecules found in biological components of a living organism, comprising its cells, tissues, and microorganisms [34]. The primary function of enzymes is to perform as biological catalysts that accelerate biochemical functions in living organisms. Enzymatic biosensors are analytical devices in which an enzyme is integrated as a bioreceptor or tightly coupled to a physical transducer to provide a discrete or continuous digital electronic/optical signal proportional to the amount of analyte in the sample [35]. Enzyme-based analytical techniques rely on detecting a co-substrate or, more precisely, the products of the catalyzed reaction [36]. Enzymes were the first specific molecular components to be used as biosensors. They continue to form the basis of much of the work published in this area. Electrochemical analysis can be performed to determine what types of enzymes are used in a biosensor and whether they function by oxidation or reduction [33].

Due to the high catalytic activity and selectivity of enzymes and the commercial availability of purified enzymes, enzyme-based electrochemical biosensors are among the most advanced and commercially successful bioanalytical devices. This is because enzymes are commercially available [37]. Electrochemical enzyme biosensors are based on detecting electroactive species after a redox reaction, either at the electrode itself or by a mediator. The application of this method resulted in the invention of the enzyme immunoassay system as well as the very well-known glucose biosensor. Both devices are used for self-monitoring blood glucose levels and were developed in a disposable format. Many types of these devices are already on the market [38]. Enzyme biosensors provide several benefits. For example, amplification of a biosensor’s response can be catalyzed by regulating the enzyme activity in response to a particular analyte, through a consistent supply of material, a wide variety of commercially available enzymes, often with well-defined and proven properties, the ability to modify the enzyme catalytic properties or substrate specificity with the use of genetic engineering [34]. The target analyte must be present in the system for the enzyme-based biosensor to accurately detect it by experiencing a change in color, composition, mass, light absorption, or emission. These changes can result in changes in the electrical or optical signals from the biosensor. The signal intensity is linearly related to the target concentration [39]. The introduction of screen-printed enzyme electrodes was critical in developing low-cost enzyme electrodes and a pen-sized meters for home blood glucose monitoring [33,40]. It offers the advantages of simplicity, portability, and a continuously operational configuration [41,42].

### 2.1. Types of Enzyme-Based Biosensors

There are so-called ‘generations’ of biosensors, varying from first-generation to fourth-generation biosensors based on enzymes. Briefly, first-generation biosensors determine the amount of analytes and/or products of enzymatic processes by determining the amount of product that diffuses to the surface of the transducer and generates an electrical response. They can also be referred to as mediatorless amperometric biosensors [43]. The first generation used oxygen as an electron donor, sensing the resulting low oxygen or free H_2_O_2_ levels [44]. The involved enzymes, known as oxidases and dehydrogenases, serve as the basis for these biosensors. Coenzymes (for example, NAD^+^, NADP^+^, NADH, NADPH, ATP FAD, and FADH) are essential for the catalysis of oxidases and dehydrogenases and must be regenerated so that the enzyme can catalyze subsequent reactions [45]. Biosensor design depends on the knowledge of the target analyte as well as on the complexity of the matrix in which the analyte must be detected. First-generation biosensors have several drawbacks, such as the technical difficulty of maintaining an airtight sample chamber and the need for a strong redox potential for the redox indicator, which can often affect the selectivity of the developed biosensor [46]. In addition, the continuous use of amperometric biosensors, especially in complex biological matrices or undiluted samples, often contaminates the transducer surface, affecting the biosensor response [47].

Glucose biosensors can be made more accurate by replacing oxygen with electron mediators such as ferrocene, ferricyanide, and quinines; these biosensors are generally less sensitive to interference from redox-active compounds, and their mediators are usually placed in membranes that block the access of potential interferents such as ascorbic acid. This type of glucose biosensor is known as one of the second generation of glucose biosensors [48]. The biosensors of the second generation are also known as mediator–amperometric biosensors. These biosensors employ mediators such as oxidants so that they may serve as electron carriers [49]. Under steady-state and flow injection conditions, second-generation electrodes that use a mediator for glucose oxidase reoxidation are more suitable for whole-blood analysis. However, the selection of mediators is an essential step. The linear range and stability of the results were better with tetrathiafulvalene, whereas dimethylferrocene needed a substantial pre-treatment [50]. To increase the analytical quality and improve the system, auxiliary enzymes and/or co-reactants are immobilized together with the analyte-transforming enzyme in second-generation biosensors [51]. However, several second-generation biosensors have problems caused by synthetic mediators’ leaking from the biosensor over time. Because of this limitation, it is impossible to incorporate soluble mediators into biosensors intended for use in vivo [52]. As a result of the immobilized mediators, second-generation biosensors are often less stable and reproducible than first-generation biosensors and are hence less attractive, which has encouraged research to discover third-generation biosensors [45].

Third-generation biosensors have a direct electrical connection between the redox center of the enzyme and the electrode, allowing a reaction to be initiated [46]. Third-generation biosensors often substitute glucose oxidase with enzymes such as glucose dehydrogenase that are better suited for direct electron transfer in place of components consisting of composites and nanomaterials (NMs) [48,53]. These biosensors are characterized by high selectivity and sensitivity because they can operate in a potential window closer to the redox potential of the enzyme, and the electron exchange between the redox center of the enzyme and the electrode occurs without a diffusion barrier due to the proximity of these two terminals [54]. In some instances, a direct electrical contact between the enzyme and the electrode may be established, considerably enhancing the electron transport efficiency. Immobilized mediators enable an effective electron transport in these third-generation biosensors, resulting in a greater current density [52]. When an enzyme and a mediator are in close proximity to the surface of the transducer, the distance that the electrons must travel is reduced, which in turn leads to faster reaction times. Immobilization prevents the mediators from escaping the biosensor film and entering the environment. This allows the sensor to be used for in vivo measurements. The applied electrode can be operated at the desired voltage, eliminating background interference. This design also allows for repeated and long-term measurements, since there is no need to replace the reagents. Ferrocene derivatives were accumulated in different types of matrices with glucose oxidase [52,55].

**Figure 2:**
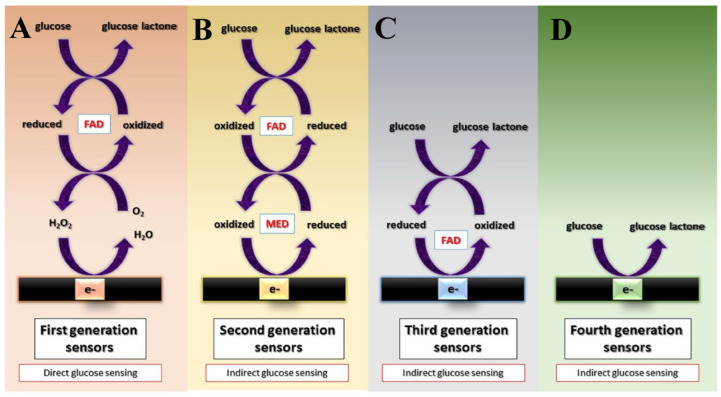
Schematic representation of four different generations of biosensors. (**A**) First generation sensors’ mechanism, (**B**) second generation sensors’ mechanism, (**C**) third generation sensors’ mechanism, (**D**) fourth generation sensors’ mechanism. “Reprinted with permission from [57]. 2021, Wiley”.

Despite all of these advancements, the successive generations of biosensors have several limitations that have not yet been thoroughly addressed, which resulted in the development of non-enzymatic glucose detection devices. These non-enzymatic glucose sensors, also known as the fourth generation of glucose sensors, depend on the principle of oxidizing glucose directly on the electrode surface [56]. Fourth-generation glucose sensors (FGGS) have been designed to enhance the glucose-sensing technology and decrease the number of intermediate stages required for glucose measurement. Diagnostic efficacy and cost-effectiveness are both improved with the use of these sensors, which are fabricated utilizing electrocatalytic copper nanostructures [57]. Schematic representation of four different generations of biosensors are shown in Figure 2.

### 2.2. Enzyme Immobilization and Its Techniques

**Figure 3:**
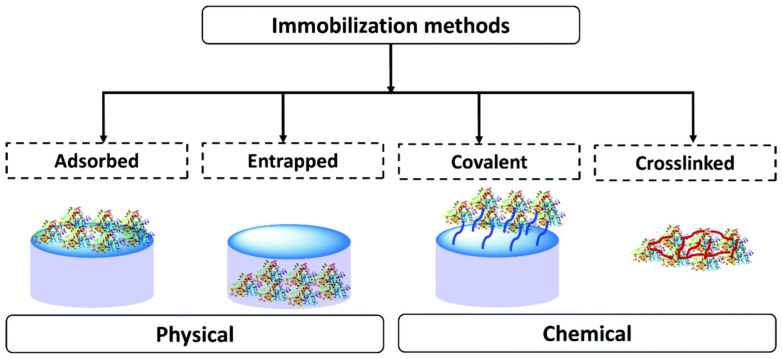
Schematic representation of the main different methods of enzyme immobilization. Reprinted with permission from [60]. 2021, RSC.

Enzyme immobilization has been shown to be an effective method to avoid the disadvantages of using free enzymes, thus improving the productivity and cost efficiency of the manufacturing process. Immobilized enzymes offer great potential for developing sensors that can detect and characterize their respective targets. It is anticipated that substrate/analyte molecules will migrate from the medium to the immobilized enzymes in enzymatic biosensors [58]. In the process of designing the section of enzyme-based biosensors that is responsible for biorecognition, the immobilization of the enzymes is an essential component. Enzyme immobilization is a crucial first step in the development of reliable biosensors that can withstand repeated applications and exhibit long shelf life, high sensitivity, high selectivity, short response time, and high reproducibility [59]. Figure 3 shows the different immobilization methods, such as adsorption, physical entrapment, covalent bonding, and crosslinking [60].

Adsorption: During the adsorption procedure, the enzyme is physically adsorbed onto a pre-prepared support material utilizing hydrophilic–hydrophobic, van der Waals, H-bonding, and/or ionic interactions [61]. In the adsorption technique, either the enzyme is applied directly to the surface of the carrier or the carrier is wholly immersed in the enzyme solution. The support is allowed time to dry, and the unbound enzymes are rinsed off the surface with distilled water or a buffer solution. Since no additional chemicals are used in adsorption, this technique is inexpensive and also has no adverse effects on the activities of the enzymes [62]. Adsorption of an enzyme onto a support is relatively simple, but the connection between the support and the protein is limited, and these materials are susceptible to leaching [60].

Entrapment: Unlike in conventional entrapment processes, the substrates and end products are not directly bonded to each other but are contained inside polymers that maintain a gap for their dispersion. The enzymes can be immobilized by a process known as entrapment immobilization, in which the enzymes are entrapped in a polymeric network or microcapsules of polymers that still allow the substrate and products to flow through [63]. Polymerization is performed in a mixture of enzymes and monomers to trap the enzymes. Entrapment, in contrast to covalent bonding, does not involve chemical contact; as a result, the enzymes are able to maintain a high level of stability while experiencing a little loss of activity [64].

Covalent binding: Covalent bonding is one of the most common methods that allow the establishment of stable complexes between enzymes and carriers. This makes it an attractive option [65]. Covalent bonding to polymer supports, a well-known form of chemical immobilization, is commonly used to immobilize enzymes in developing enzymatic biosensors. This is one of several chemical immobilization methods [66]. Enzyme stability is maintained in the presence of a solution with high ionic strength due to the strong interaction between the molecule and the support. It is most commonly applied in all cases with different functional groups to matrices with the capacity for covalent binding [67].

## 3. Nanomaterials That Are Generally Used in the Design of Enzyme-Based Electrochemical Nanobiosensors

Electrochemical enzyme-based biosensors are among the most widespread and commercially effective biosensors. The use of NMs as biosensor modification agents enhance biosensors’ sensibility and substantially limits detection, persistence, response rate, and some other analytical properties [37].

NMs play an important role in enzyme-based biosensors, boosting a sensor’s efficiency and sensitivity. These novel nanoscale materials have unique features that can considerably increase the sensing capacities of biosensors [68]. By adding NMs into enzyme-based biosensors, the surface area-to-volume ratio may be greatly boosted, allowing for the immobilization of a greater number of enzyme molecules and improving the overall enzyme–substrate interaction [69]. Furthermore, NMs have high electrical conductivity, optical characteristics, and biocompatibility, allowing for effective signal transmission and detection. Their careful control over size, shape, and surface qualities improves enzyme immobilization, stability, and selectivity, resulting in extremely sensitive and selective biosensing platforms. By enabling the quick, accurate, and accurate detection of analytes in complicated samples, the incorporation of nanomaterials in enzyme-based biosensors offers considerable potential for a variety of applications, including health care diagnosis, environmental monitoring, and food safety analyses [70,71,72]. As a result, a wide range of materials, such as carbon NMs, 2D materials, metal NPs, metal oxides, complexes, polymers, ionic liquids (ILs), and so on, are constantly being investigated. IL-based electrochemical sensors and biosensors are becoming increasingly popular due to their unique qualities, such as superior ionic conductivity, synthetic diversity, strong electrochemical stability, low toxicity, and customizable physicochemical properties [73].

**Figure 4:**
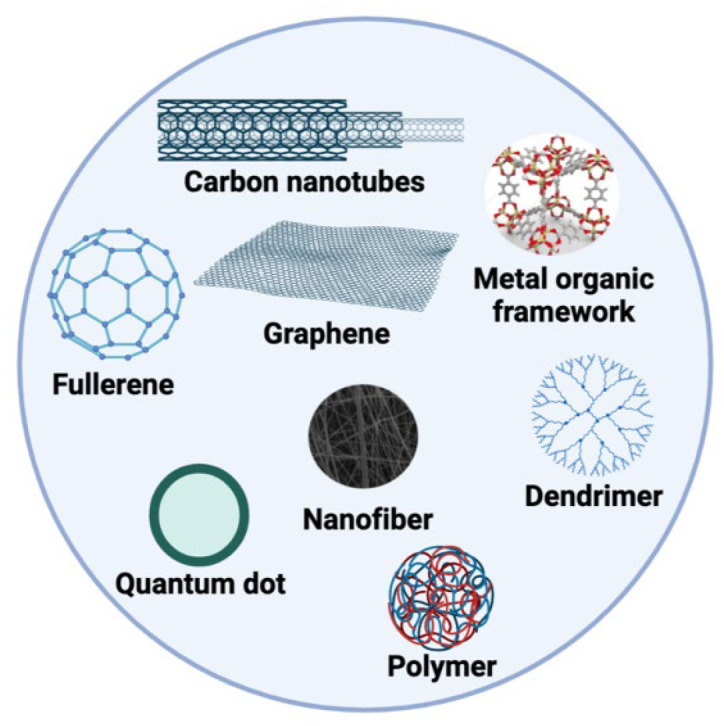
Nanomaterials that are generally used in enzyme-based electrochemical nanobiosensors.

The use of nanostructured materials of many types, each with distinctive chemical and physical properties, is a useful technology in biosensor production. NMs, which contain structural components extending in size from 1 to 100 nm, differ significantly from similar macro-scale materials [74]. Based on their chemical composition, NMs are classed as organic or inorganic. Metals and their oxides, quantum dots, metal–organic frameworks (MOFs), zeolites, and other inorganic NMs are examples of inorganic NMs; organic NMs include carbon-based NPs such as fullerenes, carbon nanotubes (CNTs), graphene and graphene oxide, fullerene, and other organic NMs, as shown in Figure 4. [75]. NMs have been utilized to regulate enzyme activity as well as enzymatic structures and functions [76]. Metal nanoparticles (NPs) were used as a transport medium to collect analytes from samples and concentrate analytes to the electrode surface to improve the analytical signal. NPs present three layers since they are not simple molecules: I (i) a surface, which may be modified with various tiny compounds, metal ions, surfactants, and polymers, (ii) a top layer, which is chemically separate from the core in every way, and (iii) a core, which is the NP’s central section and is generally referred to as the NP itself [77].

Since enzymes include several functional groups, such as carboxylic (-COOH), amino (-NH_2_), thiol (-SH), and many others, they may be easily attached directly to NPs. Adsorbents for enzymes can be NMs having hydrophobic or charged spots on their surface that can engage with enzymes or NMs with chemical groups that can bind to the matching enzyme groups [78]. Metal and metal oxide nanoparticles are the most common inorganic NMs and include ZrO_2_, TiO_2_, Fe_2_O_3_, Al_2_O_3_, CeO_2_, and MoO_3_ [79]. Metal NPs are extensively employed in biosensors because of their distinct physical and chemical characteristics. Inorganic compound-derived NPs are highly stable, very adsorptive, biocompatible, and conducive to enzyme or molecule immobilization while keeping the latter’s biological function and original structure. They have fascinating applications in both biological and chemical sensing [80].

For example, MOFs offer unique qualities, including a high yet adjustable porosity, well-defined channels or pores, and simplicity of post-synthetic modification to integrate new functional units, which makes them great candidates for sensing applications [81]. Numerous articles on the potential of MOFs for various biosensing applications have been published in recent years, but each approached them differently. While some studies focused on extremely particular forms of MOFs or composites, others did not [82], and some focused on specific sensor types [83], specific analytes [84], or specific application fields [85,86]. The advantage of MOFs over other materials is their well-defined structure, which has connecting units that are accessible to chemical modification by using a logical “design-for-purpose” approach [81].

Gold (Au) and silver (Ag) nanoparticles have the most intriguing physical characteristics for biosensing among all metallic NPs. [87]. Au and Ag NPs have emerged as promising materials for biosensors due to their unique physicochemical properties. These nanoparticles possess a high surface-to-volume ratio, excellent biocompatibility, and tunable optical and electronic properties that can be precisely tailored for various applications. These nanoparticles also offer several advantages, including easy synthesis, stability, and reproducibility [88]. Furthermore, their small size enables them to penetrate biological membranes, making them suitable for in vivo applications. Thus, Au and Ag NPs have become essential components of biosensors for a variety of biological and clinical applications. AgNPs created using bio-mediated processes may be considered cost-effective, eco-friendly, and new alternatives, with the added benefit of a reduced synthetic complexity [89]. AgNPs promote electrochemical signal amplification and improve matrix conductivity. As the surface charge of distributed AuNPs and AgNPs approaches neutralization, or a certain target–aptamer binding event happens in both nanoparticle systems, the distance between NPs decreases, and surface plasmon coupling occurs [90]. NPs with these qualities will face challenges such as bioaccumulation, toxic effects, modeling variables, regeneration, reusing, and recycling [91].

Carbon-based NMs, such as carbon nanotubes, graphene, and carbon dots, have shown a great potential for biosensors due to their unique properties. These nanomaterials offer high surface area, high conductivity, and high biocompatibility, which makes them ideal for detecting biomolecules with high sensitivity and selectivity. Additionally, the surface chemistry of carbon-based nanomaterials can be easily modified to provide specific binding sites for biomolecules, further enhancing their sensing capabilities. Overall, carbon-based nanomaterials have emerged as a promising platform for developing biosensors with improved sensitivity, accuracy, and portability, which have the potential to revolutionize various fields, including medical diagnostics, environmental monitoring, and food safety. For its good electrical conductivity, huge surface area, and ability to bind metals, polymers, and silica, graphene, a typical carbon nanomaterial, has been utilized widely as a potential electrode material for electrochemical sensors [92]. Fullerenes are unique in their low dimensionality, quantum confinement, and shape, resulting in properties that cannot be found in bulk materials. Researchers have extensively studied fullerenes’ morphology, chemical and physical properties, and functionalization possibilities. The number of publications related to fullerene research is increasing exponentially. In biomedical applications, fullerenes must be dispersed in solvents, with aqueous dispersions being preferred due to their biocompatibility, safety, and environmental friendliness. Fullerenes’ solubility in solvents expands their processing possibilities in solutions, creating uniform sheets crucial for applications such as coatings and electrodes [93].

Electrospinning is a versatile and accessible nanofiber production technique that offers various benefits, such as the ability to control fiber dimensions, a high surface area for reactions, a porous structure with interconnectivity, compatibility with a wide range of polymers, and ease of processing and functionalization. Due to these advantages, electrospinning is widely applicable in tissue engineering, catalysis, sensors, and drug delivery [22,94,95].

In electrochemical analysis, the surfactant forms an adsorption layer that raises the peak current and alters the redox potential by collecting and allocating electrons. Surfactants are increasingly used in biosensors and electrochemical sensors due to their ability to accelerate electron transport and improve analyte adsorption [96]. However, the use of surfactants in enzyme-based biosensors might have significant drawbacks. Certain surfactants could interrupt the enzymatic process or create non-specific interactions, compromising the specificity and accuracy of the biosensor. Furthermore, certain surfactants may raise cytotoxicity or immunogenicity issues, which restricts their usage in biomedical and clinical applications. Furthermore, the presence of surfactants might increase the complexity and expense of biosensor fabrication. As a result, surfactants in enzyme-based biosensors must be carefully selected and optimized to maximize their advantages while limiting any potential negative aspect [97,98,99].

## 4. Application of Enzyme-Based Electrochemical Nanobiosensors

**Figure 5:**
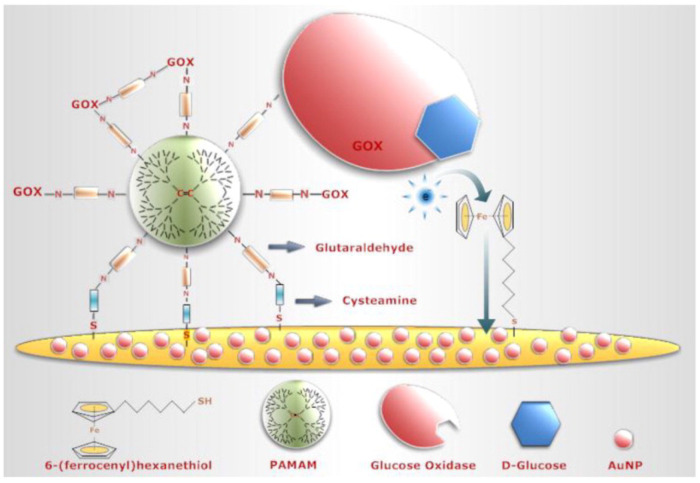
Modified gold surfaces by 6-(ferrocenyl)hexanethiol/dendrimer/gold nanoparticles as a platform for biosensing applications. “Reprinted with permission from [104]. 2013, Elsevier”.

The healthcare system applications, environmental sensing, food analysis, and security and military applications are all possible using biosensors. Enzyme-based biosensors have the potential to detect industrial pollutants and food contamination, as well as pesticides, glucose, and ethanol in food and beverages, and viral, fungal, and bacterial diseases [100]. The design of dendrimeric compounds with high thermal stability and fluorescence becomes essential for the preparation of new electroluminescent materials [101,102,103]. Among all, polyamidoamine dendrimers have generally found application in biosensors construction and cancer cell targeting due to their large number of amine groups available for biomolecule immobilization. In their study, authors have used the advantages of polyamidoamine dendrimers to increase the surface area, thus allowing the immobilization of higher amounts of glucose oxidase and the achievement of direct electron transfer between FAD on the enzyme and the electrodes [104] (Figure 5).

Another glucose biosensor, GOD/CS/PR/GCE, was suggested by Ma et al. [105] in their study, an amperometric glucose biosensor based on glucose oxidase immobilization with a layer-by-layer method and chitosan and pyrite, tightly adsorbed through electrostatic force on a glassy carbon electrode. The immobilization method provided good bioelectrocatalytic activity of GOD on the CS- and PR-modified electrode surface. The morphology and characteristics of the developed biosensor were characterized by CV, EIS, QCM-D, SEM, and AFM. Moreover, PR concentration for detecting 20 mM glucose, GOD concentrations, pH, chitosan concentration, and mediator type were optimized. The linear range of detection obtained using the designed biosensor was 0.5–60 mM, and the detection limit was 50 μM glucose. As a result, this process of using pyrite and chitosan as physically modified GOD could be helpful in designing better enzyme-based biosensors for a wide variety of practical applications [105].

Quantum dots, nanoparticles of semiconductors, were theorized in the 1970s and produced in the early 1980s. Quantum effects, which limit the energies of electrons in particles by making semiconductor materials small enough, play an important role. Quantum dots can be used in composites and solar cells that use small particles with adjustable energy levels [106,107,108].

**Figure 6:**
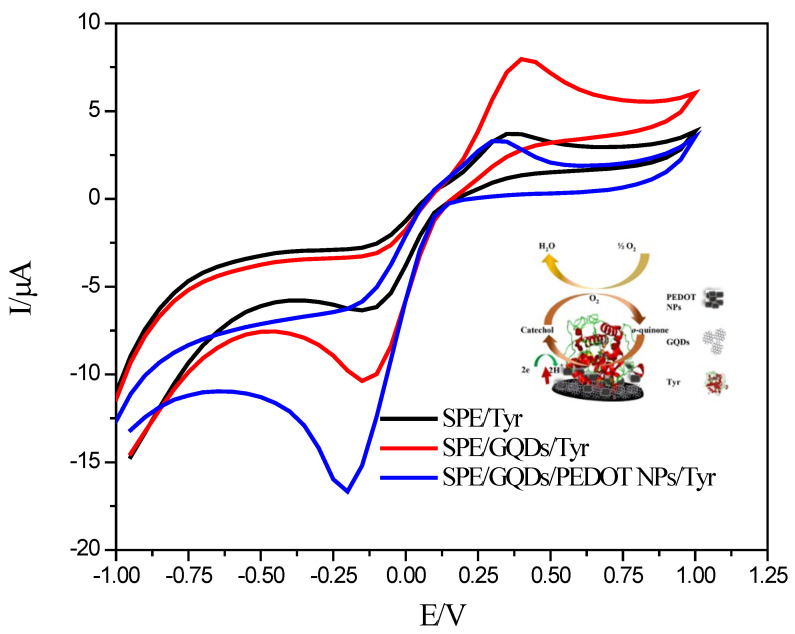
Cyclic voltammograms of SPE/Tyr, SPE/QDs/Tyr, and SPE/GQDs@PEDOT NPs/Tyr in the presence of catechol in 50 mM phosphate buffer at pH 6.5 with 0.1 M KCl. Inset: schematic representation of the designed biosensor. “Reprinted with permission from [110]. 2021, Elsevier.”

One of the crucial areas of use of nanoparticles and nanotubes is the production of nanocomposites. Nanocomposites are materials that consist of one or more different components and present the best properties of each element. The multifunctional properties of these materials extend not only to mechanical properties but also to optical, electrical, and magnetic properties [109]. A recent study was conducted by our group, focused on nanocomposites of poly(3,4-ethylene dioxythiophene) polymeric nanoparticles and graphene quantum dots in enzyme inhibition on screen-printed electrodes (SPE) [110]. In this work, the dual detection of catechol and diclofenac was carried out. To monitor tyrosinase (Tyr) enzyme inhibition by the non-steroidal anti-inflammatory drug diclofenac, the synergetic effect between the graphene quantum dots (GQDs) and polymeric nanoparticles was used, as PEDOT NPs are highly conductive polymeric nanoparticles; they significantly facilitated the electron transfer rate and improved the current response of CAT compared to SPE/GQDs/Tyr, as shown in Figure 6.

ZnSeQDs were also utilized in order to detect 17β-estradiol. Horseradish peroxidase enzyme was immobilized on the PANI:3MPA-ZnSeQDs-modified electrode surface, and a glutaraldehyde solution was used as the binder for crosslinking the HRP enzyme and the gold electrode surface (AuE/PANI:3MPA-ZnSeQDs/HRP). The electrode was modified with 3-mercaptopropionic acid-capped zinc selenide quantum dots trapped within the polyaniline framework structure. The HRP/quantum dot polymeric nanocomposite blend efficiently catalyzed the oxidation of 17β-estradiol. The influence of low and high concentrations of H_2_O_2_ on the electrochemical detection of 17β-estradiol was investigated using cyclic voltammetry. The biosensor morphology was characterized by TEM, EDX, FT-IR, UV–Vis, and EIS. The performance of the designed biosensor was evaluated using differential-pulse voltammetry. The developed biosensor’s LOD was found to be 0.2 × 10^−6^ M towards 17β-estradiol. The authors stated that the biosensor was capable of detecting 17β-estradiol concentrations in spiked tap water samples with acceptable recoveries [111]. In another study, quantum dots were utilized to detect cholesterol. A modified electrode based on ceramic-coated liposomes (cerasome), graphene quantum dots (GQDs), and polyethyleneimine (PEI) was used in the construction, for the electron transport between the enzyme and the electrode. Cholesterol oxidase (ChOx) was immobilized with a layer-by-layer self-assembled method onto the modified electrode. A ChOx/PEI/GQDs/PEI/cerasome-modified electrode was fabricated for sensitive cholesterol detection. FT-IR, TEM, and PL were used for biosensor’s cerasome-forming lipid, morphology, and GQDs characterization. Ascorbic acid, glucose, uric acid, and lactic acid were added to investigate the influence of interfering substances. The linear range of detection was found to be between 16 and 6186 μM, and the LOD value appeared to be 5 μM. The developed biosensor was further used for cholesterol detection in human blood serum, and the results were acceptable and reliable [112]. In another study, quantum dots of copper sulfide were synthesized and characterized using XRD, EDX SEM, TEM, and PL in order to be used in immobilization matrices. Then, electrodeposition of the copper sulfide quantum dots on Ti/TiO_2_ nanosheets was achieved to immobilize NADPH. The developed biosensor was used for the detection of sorbitol in serum blood to oxidize NADPH by the enzymatic reaction and reduce glucose to sorbitol. The cyclic voltammetric technique was used to monitor the sorbitol amount; the suggested biosensor was linear between 0 and 5 mM glucose concentration. A possible interference effect was also studied using fructose, mannitol, and uric acid. A real sample analysis was also performed by the authors using real blood samples taken from a hospital, with different blood glucose levels i.e., 93.00 mg/dl, 169.00 mg/dl, and 181. 000 mg/dl; using both oxidation and reduction, currents, and potentials, reliable results were obtained. The authors stated that CuS QD is a suitable immobilization candidate for developing enzyme-based sorbitol biosensors.

Moreover, in another study, the electrochemical biotransformation of 4-hydroxyphenytoin by cytochrome P450 2C19 was followed via didodecyldimethylammonium bromide-modified electrodes. The designed transducer was used as an electron donor for the reduction of the heme iron ions of the immobilized CYP2C19. The authors demonstrated the possibility of electrochemical systems based on cytochromes P450 to be applied for the detection of atypical kinetic profiles of drug metabolism [113].

Carbon-based materials such as carbon nanotubes, fullerene, carbon fibers, etc., are generally used in biosensor design due to their high stability and conductivity. In their work, an electrochemical biosensor based on immobilizing the GOx enzyme on in situ-grown carbon nanotubes on a gold microelectrode array fabricated on a glass substrate (CNTs/Au MEA) was used for detecting glucose. Cyclic voltammetry and electrochemical impedance spectroscopy were used to detect glucose. SEM, EDX, and HPLC were used for surface characterization. A three-electrode system was utilized using modified CNTs/Au MEA as the working electrode, platinum wire as the counter electrode, and Ag/AgCl as the reference electrode. Glucose concentration was found to be between 0.2 and 27.5 μM. Moreover, the proposed sensor showed an LOD of 0.2 ± 0.0014 μM. The H_2_O_2_ amount was analyzed to observe the catalytic reaction of β-D-glucose and the GOx enzyme with a conventional colorimetric technique. The interference of ascorbic acid, citric acid, cysteine, uric acid, urea, dopamine, cholesterol, and paracetamol was also studied. Using the designed biosensor, all the 64 analyzed samples could individually be used for the detection of glucose, and the process could be monitored, which is the main advantage of using this biosensor. The developed biosensor (p-PDA)/CNTs/Au MEA possesses acceptable characteristics such as high detection sensitivity, exceptional reproducibility, and excellent shelf life [114]. In another study, Ag, reduced graphene oxide, and chitosan were used to fabricate a nanocomposite-based biosensor, and acetylcholinesterase enzyme was immobilized on the Ag/rGO/CS matrix. The developed Ag/rGO/CS/AChe biosensor exhibited a good electrocatalytic effect for determining carbaryl pesticides. Glutaraldehyde as a crosslinker was added to the Ag/rGO/CS nanocomposite to increase the immobilization ability of the AChE enzyme. AChE catalyzes the hydrolysis of acetylthiocholine chloride, and based on the presence of the carbaryl pesticide, which inhibits the activity of AChE, a decrease in the ATCl response occurred. Using the designed Ag/rGO/CS/Ache biosensor, the linear range of detection was between 1.0 × 10^−8^ and1.0 μg mL^−1^, and the limit of detection was found to be 1.0 × 10^−9^ μg mL^−1^. According to these results, the Au-Ag/rGO/CS@AChE biosensor allows acceptable result reproducibility and has long-term stability.

**Figure 7:**
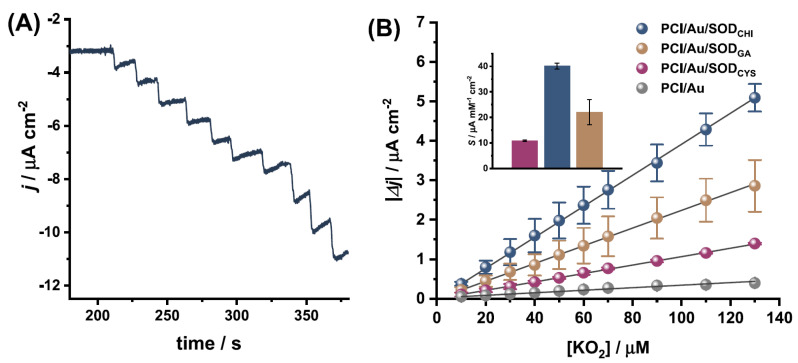
(**A**) The typical fixed potential chronoamperometric response obtained in 0.1 M PB, pH 8.0, at −0.3 V using the PCl/Au/SODCHI biosensor with successive additions of KO_2_, (**B**) calibration curve constructed from the amperograms obtained at −0.3 V for each SOD biosensor construction; (inset, corresponding sensitivity values). “Reprinted with permission from [115]. 2022, Talanta.”

In their study, Sanz et al. fabricated disposable superoxide dismutase (SOD) biosensors for the detection of superoxide (O_2_^•−^) in cell culture media. Superoxide dismutase was immobilized on gold metalized polycaprolactone electrospun polymeric fibers (PCl/Au). Three methods were used for immobilization, i.e., cross-linking with EDC/NHS at a self-assembled cysteine monolayer (PCl/Au/SOD_CYS_), biopolymer encapsulation with chitosan (PCl/Au/SOD_CHI_), and cross-linking with glutaraldehyde (PCl/Au/SOD_GA_) (Figure 7). PCl/Au/SOD_CHI_ and PCl/Au/SOD_CYS_ were evaluated. H_2_O_2_, uric acid, dopamine, and ascorbic acid were studied for interference effects in the chosen biosensors. Cyclic voltammetry (CV) was used to examine the electroactive area of the developed biosensor, and SEM was used to evaluate the three biosensors’ surface morphologies. The biosensors were characterized by fixed potential amperometry in the presence of an increasing concentration of O_2_^•−^. The analytical parameters and the effect of the immobilized SOD concentration on the biosensor response were evaluated. Moreover, storage stability was also evaluated by recording the biosensor response after 1 month of storage in a fridge and in air. Interestingly, the PCl/Au/SOD_CYS_ biosensor had the highest sensitivity of 16.1 μA mM^−1^ cm^−2^ for superoxide monitoring in cell culture media. The LOD was found to be 1.9 µM for PCl/Au/SOD_CYS_, with a linear range of detection of 20–100 μM [115].

Three-dimensional nanoporous gold (NPG) was also used in electrochemical biosensor design due to its wide surface area, high conductivity, and good electrocatalytic activity for the detection of glycerol using coimmobilized glycerol kinase and glycerol-3-phosphate oxidase in thioctic acid, linked to NPG by covalent binding. Chitosan was encapsulated to further prevent the enzymes from falling off. In their work, combining covalent bonding and embedding methods to immobilize the enzymes, the authors stated that improving the catalytic performance of the examined enzymatic biosensor was one of the exciting points of this study. SEM was used to characterize the structure of the prepared materials. Cyclic voltammetry and electrochemical impedance spectroscopy were used to evaluate the electrochemical performance of the designed biosensor. The authors optimized the pH of PBS from 6.0 to 8.0.

Moreover, they studied the long-term stability of the GK/GPO/CHIT/TA/NPG/AuE biosensor. Glucose, ethanol, ascorbic acid, urea, and citric acid were added to observe how they influenced the glycerol response. The reported biosensor’s sensitivity was 9.17 μA mM^−1^, the linear range of detection was found to be between 0.1–5 mM, and the detection limit was 77.08 μM. The authors indicated that GK/GPO/CHIT/TA/NPG/AuE biosensors could be used to determine glycerol in real samples, such as red and white wine samples [116].

Moreover, conducting polymers, such as polyaniline, polypyrrole, etc., are important in enzyme-based biosensor design [117,118]. To illustrate this, Lou et al. immobilized laccase in a polyaniline/magnetic graphene composite electrode (PANI/MG-Lac-GCE) and used it for the detection of hydroquinone. The electrochemical properties of the modified electrodes were analyzed, and the biosensor’s performances were evaluated. Finding an assembly method that can provide a robust and reproducible electrode is a key difficulty in nanostructure-based working electrode production for biosensing systems. When employing electrochemical methods to detect biomarkers, the nanoparticles must establish a strong bond with the electrode surface to avoid the development of corrosion on the modified working electrode as a result of the applied potential and voltage. Therefore, developing a reliable production process is critical [9]. A three-electrode system was utilized, i.e., a modified glassy carbon electrode as the working electrode, a Pt electrode as the counter electrode, and a saturated Ag/AgCl electrode as the reference electrode. Cyclic voltammetry was used to evaluate the working electrodes by observing the relationship between potential and current and the peak value of the current. The chronoamperometric method was used to evaluate the biosensors by recording the relationship between substrate concentration and current. These two methods were applied to evaluate the electrochemical performance of the biosensors in the detection of hydroquinone. The selectivity was found to be 0.03639 A/(M) using the suggested biosensor. Moreover, the linear range of detection was between 0.4 and 337.2 μM, and the detection limit was calculated to be 2.94 μM. Its electrocatalytic effect on hydroquinone proved that the prepared biosensor is a prospective phenolic biosensor for real water applications [119]. In a recent study by our group, conducting polymers of benzoxadiazolecore and thienopyrroledione were coupled with benzodithiophene. The thus obtained three random copolymers with fullerene (C60) were used for an enzyme immobilization matrix for tyrosinase (Tyr). This was the first fabricated biosensor for the inhibition of Tyr by indomethacin, a pharmaceutically active compound. The developed biosensor GE/poly[BDT-alt-(TP;BO)]-C60/Tyr was used as a working electrode, Ag/AgCl was used as a reference electrode, and a platinum wire was used as an auxiliary electrode. CV, SEM, AFM, EIS were used to observe the properties of the finalized modified surfaces. The effects of the biosensor parameters on biosensor response, copolymer composition, ratio optimization, inhibition time, and inhibitor concentration were optimized. The linear range of detection of the suggested biosensor was found to be 0.5–62.5 μM, and the LOD was found to be 0.11 μM [120].

Ma et al. also suggested a tyrosinase-based biosensor for the detection of bisphenol A (BPA). Tyrosinase was immobilized layer-by-layer in an ultrathin copper–porphyrin MOF (metal–organic framework) nanofilm, and an ultrasensitive biosensor (Tyr@Cu–TCPP/GCE) was developed to detect bisphenol A rapidly. Moreover, the authors compared the electrocatalytic activity of biosensors based on Tyr, Tyr/Cu–TCPP, and Tyr@Cu–TCPP for BPA determination. SEM, TEM, FTIR, UV-1700, XRD, XPS, and AFM were used to characterize the electrochemical morphology of the modified electrode. The Tyr@-Cu–TCPP/GCE biosensor was used to detect BPA in milk and plastic mineral water bottle samples. The developed biosensor’s stability and sensitivity were investigated under varying conditions, such as different storage times, high temperature, and strong acidity/basicity. The biosensor’s LOD was found to be 1.2 nM, and the linear range of detection was found to be between 3.5 nM and 18.9 M [121].

In another study, Ivanov et al. investigated the reversible inhibition properties of donepezil and berberine towards the acetylcholinesterase enzyme. The acetylcholinesterase enzyme was physically entrapped in polyelectrolyte complexes, and the authors stated that hydrophobic π-staking interactions with aromatic residues also contributed to the inhibition complex formation. Hence, the developed biosensors showed inhibition towards donepezil and berberine with a limit of detection between 0.46 and 70 nM [122].

The sequential enzyme biosensor using glucoamylase (GAm) and glucose oxidase (GOx) co-displayed on yeast recombinants was used to detect starch and glucose by Liu et al. The recombinants yeast–GAm&GOx with different GAm/GOx ratios were fabricated as biocatalysts on the reduced graphene oxide (RGO)-modified glassy carbon electrode (GCE). Then, the yeast–GAm&GOx (2:1)/RGO/GCE, yeast–GAm&GOx (3:1)/RGO/GCE, and yeast–GAm&GOx (1:1)/RGO/GCE biosensors were fabricated and evaluated. According to the overall catalyzing rate of the yeast–GAm&GOx (2:1)/RGO/GCE, the biosensor was subsequently efficient in the linear range of detection of 50–3500 mg/L for starch and in that of 2.0–100 mg/L for glucose. The authors examined possible interference with other saccharides such as sucrose, xylose, lactose, galactose, mannose, arabinose ribose, as well as with ascorbic acid. The suggested biosensor was not affected by any of the interference factors. Thus, yeast-GOx/RGO/GCE could be used for sensitive starch and glucose detection in real samples [123]. Some selected studies about enzyme-based electrochemical nanobiosensors are tabulated in Table 1.

## 5. Conclusions

Electrochemical biosensors, whose working principles are based on the electrochemical characteristics of the analyte and the transducer, are the most commonly researched and utilized biosensors. Electrochemical biosensors have great sensitivity, selectivity, and detection capabilities. The future of enzyme-based biosensors looks promising in a variety of research and application areas. Enzymes are used as the biorecognition element in these biosensors to detect and quantify certain analytes of interest. One of the major benefits of enzyme-based biosensors is their excellent selectivity and sensitivity, which allows for the detection of target molecules with outstanding precision and low detection limits. Furthermore, enzymes are adaptable and may be programmed to be used in a wide range of applications, including medical diagnostics, food safety monitoring, environmental sensing, and bioprocess control. The electrochemical biosensors multidisciplinary study area connects basic science concepts (physics, chemistry, and biology) with foundations of micro/nanotechnology, electronics, and applied medicine. Electrochemical biosensors are analytical devices that convert biochemical events such as enzyme–substrate reactions and antigen–antibody interactions into electrical signals such as current, voltage, impedance, etc. Among all biosensors, enzyme-based electrochemical biosensors have been generally used in various applications in recent years. Furthermore, enzyme-based biosensors frequently have fast reaction times, allowing for real-time monitoring and analyses. These biosensors, however, have several drawbacks. Changes in temperature, pH, and other environmental factors can influence the stability and activity of the enzymes, affecting the reliability and repeatability of the readings. Additionally, the cost of the enzymes and their immobilization onto appropriate transducer surfaces might be prohibitively expensive, impeding the large-scale commercialization and widespread use of these biosensors. Current advances in enzyme engineering, NMs, and immobilization methods are expected to overcome these constraints, making enzyme-based biosensors an important tool in future analytical science and biotechnology. As nanomaterial synthesis, design, and applications are further improved, enzyme immobilization techniques and the biosensing capability of enzyme-based research will certainly also increase, and investigations will continue in this area.

## Figures and Tables

**Table 1:** Some selected enzyme-based electrochemical nanobiosensors.

Nanomaterial	Target	Enzyme	Support	Detection Principle	LOD	Application	Ref.
PCL:PAA/PAMAM/PyOx	Glucose	PyOx	GCE	CV, DPV and EIS	0.0085 mM	Artificial serum, urine, salvia, sweat	[95]
Au/AuNP/(FcSH + Cyst)/PAMAM/GOx	Glucose	GOx	AuE	CV and AMP	0.6 mM	Cherry juice Fizzy	[104]
AuE/PANI:3MPA-ZnSeQD/HRP	17β-Estradiol	HRP	AuE	CV and DPV	0.2 × 10^−6^ M	Wastewater	[111]
CNT	Glucose	GO_x_	Au MEA	CV and EIS	0.2 ± 0.0014 µM	Blood serum samples	[114]
PANI/MG	hydroquinone	Lac	GCE	CV and CA	2.94 μM	Actual water sample	[119]
Cu–TCPP	Bisphenol A	Tyr	GCE	DPV	1.2 nM	Milk and plastic mineral water	[121]
AuNPs	polyphenols	lac	SPCE	CV	0.83 μM	Polyphenols in propolis	[124]
TiO_2_ NT	H_2_O_2_	HRP	PEC electrode	photoelectrochemical	0.7 nM	NS	[125]
AuNPs	glyphosate	urease	Ion selective electrode (Elite 8051)	potentiometry	0.5 ppm	Pesticides	[126]
Mucin and CNT	Glucose	Albumin	Pt	AMP	3 μM	Human plasma	[127]
GO/Fe_3_O_4_	Glucose	GO_x_	GCE	AMP	106.5 μA mM^−1^	Health	[128]
Ferrite NPs	Urea	Urease	GCE	CV and DPV	0.17 µM	Soil and milk samples	[129]
PEDOT-GONs	Catechol	Lac	GCE	CV	0.032 μM	Real water samples	[130]
Brushite cement-GA	Tyramine	PPO	GCE	CV	4.85 × 10^−8^ M	Gouda and brie cheeses	[131]
Sol–gel/Au-NF/MWCNT	D-alanine	DAAO	GCE	LSV and CV	20 nM	Human serum	[132]
MOF	Hydrogen peroxide	HRP	GCE	AMP and CV	0.09 μM	NS	[133]
ERGO-MWCNTs	Catechol	Lac	GCE	CA	0.3 μM	Fruit juice samples	[134]
	Pyrogallol						
	Epicatechin						
	Gallic acid						
	1,2-dihydroxybenzoic acid						
	Caffeic acid						
	Chlorogenic acid						
	Rutin						
	Catechin						
	Dopamine						
Chitosan/ZnO	Melamine	AChE	PtE	CV	3 pM for Melamine 1 pM for urea	Adulterated milk samples	[135]
	Urea						
ND-PS	Catechol	Tyr	GCE	DPV	3.9 × 10^−7^ M	River and tap water	[136]
Cu-MOF/CS/Pt	Lactate	LOx	SPCE	CA	0.75 μM	Sweat, saliva, red and white wines	[137]
PdPt NPs/Ch-IL/Gr-MWCNTs-IL	Tyrosine	TyrH	GCE	DPV	0.009 × 10^−9^ M	Cheese, egg and yogurt	[138]
Au NPs/Chitin-IL/PEDOP/Gr-MWCNTs-Fr-IL	Cholesterol	ChO, ChE and HRP	GCE	CA	0.07 μM	Rat plasma	[139]
rGO-AgNPs/Gr	L-dopa	PPO	GCE	CA	1.85 μM	Urine	[140]
rGO	Carbamate	AChE	GCE	DPV	1.9 nM	Tomato	[141]
Fe_3_O_4_@Au/(MnO_2_)	Glucose	GOx- ADH	CPE	CA	0.1 mM for glucose and 60 mM for ethanol	Honey wine fermentation with wine yeast (*Saccharomyces cerevisiae* Type II)	[142]
	Ethanol						
Fe_3_O_4_/PPy@ZIF-8	Glucose	GOx	GCE	CA	0.333 μM	Serum	[143]
Poly(L-Asp)/MWCNT	Xanthine	XO	GCE	DPV	3.5 × 10^−4^ μM	Fish meat	[144]
Pt@UiO66-NH_2_	Organophosphorus pesticides	AChE	GCE	DPV	4.9 × 10^−15^ M	Cabbage and apple	[145]
PLLY/CiA-GR	17β-estradiol	Lac	GCE	DPV	0.13 pM	Human urine	[146]
IL/GCE and GO-IL/GCE	Choline	AChE-ChO	GCE	ADPSV	0.885 nM	Human serum	[147]
	Acetylcholine				1.352 nM		
POxNPs/AuE	Pyruvate	POx	AuE	CA	0.67 μM	Serum	[148]
GKNPs/GPONPs/GrONPs/	Glycerol	GPO	PGE	CA	0.002 μM	Blood serum	[149]
GrO/AuNPs/PVA/HFB1	Pyruvate	Lactate dehydrogenase	GCE	DPV and CA	8.69 nM	Serum	[150]
AuNP-PANSA	Tyramine	Tyr	GCE	CA	0.71 μM	Fermented food and beverages	[151]
PBCB_ethaline_-HNO_3_^PTD^/MWCNT	Dichlorvos	ChOx	GCE	CA	1.6 nM	Orange juice	[152]
MNPs/IrOxNPs	Methimazole	Tyr	SPE	CA	0.006 μM and 0.003 μM	Human serum and pharmaceutical dosage form	[153]
(poly(BODT-co-FMOC)	Donepezil	AChE-ChO	GE	CA	0.027 μg/L for Donepezil	Tap water	[154]
	Neostigmine				0.559 μ/L for Neostigmine		
ERGO/IrOxNPs	Captopril	Tyr	SPE	CA	0.008 μM and 0.019 μM	Human serum and pharmaceutical dosage form	[155]
CTAB-NCC/QDs	Phenol	Tyr	SPCE	DPV	0.082 μM	Lake water	[156]
	Catechol				0.125 μM		
	o-Cresol				0.007 μM		
	4-Chlorophenol				0.021 μM		
Nafion/ZnO QDs	Uric acid	Uricase	SPE	CA	22.97 ± 10 μM	Urine samples	[157]
GQDs-AuNPs/PDDA-MWCNTs/CS/CBA	glucose	GOx	C-BPE	C-BP-ECL	64 nM	human serum samples	[158]
PAN-MWCNTs/PEDOT	glucose	GOx	Pt disk electrode	AMP	2.30 μM	Blood serum samples	[159]
PAN-MWCNTs/PPy					2.38 μM		
ZIF-8/CaCO_3_ NPs	glucose	GOx	Bare Au electrode	EIS	NS	Real honey samples	[160]
Pt-HEC/LSG	Glucose	GOx	Pt	CV and EIS	0.23 μM	In human sweat	[161]
SPCE/HRP and SPCE/PB/HRP	Caffeic acid	HRP	SPCE and SPCE/PB	CV	0.9 μM	In food supplements	[162]
GCE/Fe_3_O_4_@graphene/Ab/Lac	Free thyroid hormone	Lac	GCE	CV	45.9 nM	In synthetic serum samples	[163]
enzyme-Cu_3_(PO_4_)_2_/CC	Glucose	GOx	GE	CV	2.05 μM	NS	[164]
C-MWCNT/DAO/EDC-NHS/GA	Cadaverine	DAO	SPE	Voltammetry and DPV	0.8 μg/mL	Stock solutions and artificial salvia	[165]
NiMn-LDH-MOF/GCE	Glucose	GOx	GCE	CV	0.87 μM	In actual serum samples	[166]
AChE-Cu_3_(PO_4_)_2_ HNF/Apt/AuNP/CP	isocarbophos	AChE	CP	CV-EIS- SWV	0.016 pM	Real agricultural samples (oilseed rape, cabbage, apple, and pear)	[167]
	dichlorvos				0.028 pM		
	methamidophos				0.071 pM		
	parathion				0.113 pM		
SBA−15/APTES/GA/LOx mini-reactor connected in front of the AgA-SPE	L-lactic acid	LOx	SPE	AMP	12.0 μmol L^−1^	In wine and daily products	[168]
AuNPs/TA-APTES/aCC	Glucose	GOx	SPE	CV and EIS	3.3 μM	In sweat sample	[169]
Ag/AgCl, Pt electrode and Pt wire	Lactate	LOx	Glassy electrode	CV and chronoamperometry	31 μM	Lactate produced by foodborne lactic acid bacteria in real samples	[170]
AChE/Ag@CuO/PANI/ITO	Paraoxon-ethyl	AChE	ITO	CV	11.35 pM	Banana, tomato and soil samples	[171]
ChBD-GluOx/PB/SPC	Glutamate	GluOx	Pt electrode	CV	53.4 µA L mmol^−1^ cm^−2^	Food ingredient	[172]
Nafion/GOx/GF	Dopamine	GOx	GCE	CV	0.6 μM	Biological samples	[173]
	Glucose				0.41 μM		
GluOx/PMPD/Pt/GRE	Glucose	GluOx	GRE	CV	0.536 μM	Cucumber fruit and juice	[174]
AChE- CS/GP-AuNP-PEDOT:PSS/SPCE	Chlorpyrifos	AChE	SPCE	DPV	0.07 nM	In real cabbage sample	[175]
Fe_3_O_4_@COF	Hydroquinone	HRP	GCE	DPV	0.12 μM	Environmental water samples	[176]
GO/nafion/GCE	Glutathione	GSH-Px	GCE	DPV	1.5 nM	Hemolyzed erythrocyte, dextrose saline, tablet	[177]
Polystyrene/rGO-MNP-PDA/Anti-CRP	CRP	GO	SPCE	CV, DPV and EIS	0.33 ng/mL	Artificial saliva	[178]
3-APBA	Catechol	Tyr	Au SPE GCE	CV	0.25 μM	Green tea	[179]
PdCu	Lactate	LOx	LIG	AMP	0.28 μM	NS	[180]
PPy-IC-DS1-AuNP	Carbaryl	AChE	ITO	AMP	0.033 ng cm^2^ mL^−1^	Tap water	[181]
PEDOT:PSS/Ti_3_C_2_/GQD	Glucose	GOx	SPE	DPV	65 µM	NS	[182]
β-CD-AuNPs	Catechol	Tyr	GE	AMP	0.42 μM	Drug Inhibition	[183]
Pt/PPy/Chi	Atch	AChE	Pt	DPV	0.45 µM	NS	[184]
	Paraoxon				0.17 nM		
Chi/Ti_3_C_2_T_x_	Cholesterol	ChOx	GCE	DPV	0.11 nM	Human serum	[185]
Ti_3_C_2_/Nafion film	Hydrogen peroxide	HRP	GCE	DPV	1 µM	Human serum	[186]
ZnONPs-ATP-GO	Sucralose	Lac	GCE	DPV	0.32 μM	Food samples	[187]
Cellulose acetate–CS/GOx	Glucose	GO	GCE	CV	4.8 µM	Artificial tears, urine, sweat and serum	[188]
PVA-PEI/MNP/GO	Glucose	GO	SPE	CV and EIS	11.5 μM	Synthetic spiked samples	[189]
Ant-PAA/Lac	Phenol	Lac	GCE	CV and EIS	0.046 mM	Artificial wastewater	[190]
PCL-Chi/PAMAM-Mt	U87		GCE	CV, DPV and EIS		NS	[191]
PVA/PAMAM-Mt	Glucose	PyOx	GCE	CV, DPV and EIS	0.7 µM	Soft drink cola	[192]
Cysteamine/PAMAM	Glucose Ethanol	PyOx AOx	AuE	FIA-AMP	NS	Fermentation broth	[193]
AG/PyOx/CHIT–CNT AG/PyOx/CHIT	Maltose	PyOx α-glucosidase	GE	AMP	NS	Beer samples	[194]
PAMAM/Cyst/AOx	Ethanol	AOx	AuE	AMP	0.016 mM	Alcoholic beverage and yeast cultivation samples	[195]
CP/AuNP/GOx	Glucose	GOx	GE	CV	0.0021 and 0.0063 mM	Fizzy with orange Fizzy coke Lemonade Ice tea Peach juice Green tea Orange juice	[196]
CNTPE	Glucose	PyOx	CPE	CV	NS	In wine samples	[197]
AuNP/PANI/AgCl/Gelatin	Glucose	PyOx	GCE	CV and DPV	NS	Coke Lemonade Green tea Fruit juice Red Bull White wine Red wine	[198]
PDA/Cyst/AuNP	Glucose	GOx PyOx	AuE	FIA-AMP	38.97 µM 1.27 µM	Yeast fermentation	[199]
PEG/AuNP/GOx	Glucose	GOx	PtE	CV	0.06 mM	Ice tea Cherry juice	[200]
FDH/PAMAM	Fructose	FDH	AuE	CV	NS	Fruit juices Fizzy energy drink	[201]
Pt/MoO_3_/GCE/GOx	Glucose	GOx	GCE	CV	0.025 mM	NS	[202]
PyOx–IL–Pt–MnOx/GCE	Glucose	PyOx	GCE	CV	2.0 μM	Coke Orange juice	[203]
PGA–Mt/PyOx	Glucose	PyOx	GCE	AMP	1.2 μM	Coke and other fizzy drinks	[204]
MNP-His/Cu/Lac	Phenol	Lac	CPE	CV	NS	In culture medium	[205]
MNP/GOx	Glucose	GOx	CPE	AMP	NS	Fruit juices	[206]

3-APBA:3-aminophenylboronic acid; 3MPA: 3-mercaptopropionic acid; Ab: antibody; Acc: activated carbon cloth; aCC: activated carbon cloth; AChE: acetylcholinesterase specific binding aptamer inorganic; ADH: alcohol dehydrogenase; ADPSV: Anodic differential pulse stripping voltammetry; AG: α-glucosidase; AgA-SPE: Amalgam-SPE; AgCl: silver chloride; Ant: anthracene; AMP: amperometry; Apt: aptamer; APTES: 3-aminopropyltriethoxysilane; Asp: aspartic acid; Au MEA: Gold microelectrode arrays; AuE: gold electrode; AOx: alcohol oxidase; AuNP: gold nanoparticles; BODT: 5,6-bis(octyloxy)-4,7-di(thiophen 2-yl)benzo[c][1,2,5]oxadiazole; BODIPY: 4,4difloro-4-bora-3a,4a-diaza-s-indacene; CA: chronoamperometry; CiA: critic acid; CAT: catalase; CBA: C-BPE anode; C-BPE: closed bipolar electrode; C-BP-ECL: closed bipolar electrochemiluminescence; CC: carbon cloth; ChBD: Chitin-binding domain; ChE: cholesterol esterase; Chi: chitosan; ChO: cholesterol oxidase; ChOx: choline oxidase; CNT: carbon nanotubes; CNTPE: CNT-modified carbon paste electrode; CRP: C-reactive protein; COF: covalent organic frameworks; CP: carbon paper; CPE: carbon paste electrode; CS: Chitosan; CTAB: cetyltriammonium bromide; CP: conducting polymers; CPE: carbon paste electrode; Cyst: cysteamine; CV: cyclic voltammetry; DAAO: D-amino acid oxidase; DAO: diamine oxidase; DPV: differential pulse voltammetry; DTP-Trp: dithione [3,2-b:2′,3′-d] pyrrole- tryptophan; DS: dodecyl sulphate; EDC:1-ethyl-3-(3-dimethylaminopropyl)carbodiimide hydrochloride; EIS: electrochemical impedance spectroscopy; ERGO: electrochemical eduction of graphene oxide; FeMOFs: Fe (III) metal organic frameworks; FcSH: 6-(Ferrocenyl) hexanethiol; FIA: Flow injection analysis; FMOC: (2-(((9H-fluoren-9-yl)methoxy)carbonylamino) acetic acid; Fr: 1,1′-ferrocenedicarboxylic acid; FDH: fructose dehydrogenase; GA: glutaraldehyde; GCE: glassy carbon electrode; GE: graphite electrode; GF: graphene film; GK: glycerol kinase; GluOx: glutamate oxidase; GO: graphene oxide; GONs: graphene oxide nano-sheets; GOx: glucose oxidase; GP:Graphene; GPO: glycerol-3-phosphate oxidase; GQDs: graphene quantum dots; GR: graphene; Gr: graphite; GRE: graphite rod electrode; GrO: graphene oxide; GSH-Px: glutathione peroxidase; Hb: hemoglobin; HEC: hydroxyethyl cellulose; HFB1: hydrophobin; HNF: hybrid nanoflowers; HRP: horseradish peroxidase; His: histidine; IC: indigo carmine; IL: ionic liquids; IrOx: iridium oxide; ITO: Indium doped tin oxide; L1AN: lactose 1-aminonaphthalene; Lac: laccase; LDH: layered double hydroxide; LIG:Laser induced graphene; LOx; Lactate oxidase; LSG: laser-scribed graphene; LSV: linear sweep voltammetry; MG: magnetic graphene; MNP: magnetic nanoparticle; MnOx: manganese oxide; MOF: metal–organic framework; MoO_3_: molybdenum oxide; MWCNT: multiwalled carbon nanotubes; Mt: montmorillonite; NCC: nanocrystalline cellulose; ND: nanodiamonds; NHS: N-hydroxysuccinimide; NP: nanoparticles; NS: nanosheet; NS: not stated; PAA: poly(amic) acid; PAN: polyacrylonitrile; PANI: Polyaniline; PANSA: poly(8-anilino-1-naphthalene sulphonic acid); PAMAM: poly(amidoamine); PB: Prussian blue; PBCB: poly(brilliant cresyl blue); PCL: poly-Ɛ-caprolactone; PAA: poly(acrylic) acid; PDA: polydopamine; PDDA: poly(diallyldimethylammoniumchloride); PEC: photoelectrochemical; PEG: poly(ethylene glycol); PEDOP: poly(3,4-ethylenedioxypyrrole); PEDOT: poly(3,4-ethylenedioxythiophene); PEI: polyethyleneimine; PGA: polyglycolide; PGE: pencil graphite electrode; PtE: platinum electrode; PLLY: poly-L-lysine; PMPD: poly(m-phenylenediamine) film; PO: paraoxonase; POx: pyruvate oxidase; PPO: polyphenol oxidase; PPy: polypyrrole; PS: potato starch; PSS: poly(styrenesulfonate); PTD: potentiostatic deposition of polymer film; PVA: Polyvinyl alcohol; QDs: quantum dots; PyOx: pyranose oxidase; rGO: reduced graphene oxide; SBA−15: mesoporous silica powder; SPC: screen-printed nanocube; SPCE: Screen-printed carbon electrode; SPE: screen-printed electrode; SWV: square wave voltammetry; TA: Tannic acid; TCPP: tetrakis(4-carboxyphenyl)porphyrin; Ti_3_C_2_T_x_:MXene; Tyr: tyrosinase; TyrH: tyrosine hydroxylase; XO: xanthine oxidase; ZIF: zeolitic imidazolate framework; ZNO: zinc oxide; ZnSe: zinc selenide; ZnSeQD: zinc selenide quantum dots; β -CD: β-cyclodextrin.

## Data Availability

Not applicable.
